# Symptoms and Risk Factors for Long COVID: A Cross‐Sectional Study in Primary Care

**DOI:** 10.1002/jmv.70579

**Published:** 2025-08-29

**Authors:** Assel Muratovna Shigayeva Ferreira, Flávia Emília Leite de Lima Ferreira, Caio César Ferreira Alverga, João Agnaldo do Nascimento, André Luís Bonifácio de Carvalho, Gabriel Rodrigues Martins de Freitas, João Aurílio Cardoso de Moraes, Izabele da Silva Rocha, Lucas Tomaz da Silva, Beatriz Carolinny Pereira da Silva Alves, Cleidilaine Ramos de Oliveira, José Ricardo Araujo Cardoso, Ruth Maria Mendonça Anacleto, Leandro Pernambuco

**Affiliations:** ^1^ Center of Medical Sciences Federal University of Pernambuco Recife Brazil; ^2^ Center of Health Sciences Federal University of Paraiba Joao Pessoa Brazil; ^3^ Center of Medical Sciences Federal University of Paraiba Joao Pessoa Brazil

**Keywords:** COVID‐19, long COVID, primary care, risk factors

## Abstract

This study aimed to determine the occurrence and risk factors for persistent symptoms after mild to moderate severe acute respiratory syndrome coronavirus 2 (SARS‐CoV‐2) infection in patients who presented in primary care during the 2021 pandemic. A retrospective cross‐sectional survey was conducted in six public family health care units in Joao Pessoa, Brazil. A questionnaire with a set of 18 validated clinical outcomes was used to assess prolonged symptoms beyond 3 months of duration in 226 adults who had confirmed SARS‐CoV‐2 infection. Binary logistic regression models were used to estimate adjusted odds ratios (aOR) and risk factors for Long COVID. A total of 16 outcomes were significantly associated with Long COVID. The largest aOR were estimated for short‐term memory loss, anxiety, and loss of attention. The risk factors for Long COVID included ≥ 5 symptoms (15.82, 7.33–34.15, *p* < 0.0001), female sex (aOR: 1.91, 95% CI: 1.03–3.53, *p* = 0.032), age 40–49 years (3.45, 1.14–10.51, *p* = 0.029), and age 70+ years (4.0, 1.01–15.51, *p* = 0.045). Findings highlight a high frequency of persistent symptoms in adults who initially had non‐severe SARS‐CoV‐2 infection, who are predominantly of working age, and who did not present multiple comorbidities. This study supports the need for assessing clinical outcomes and risk factors on Long COVID in primary care using routinely recorded clinical outcomes.

## 1 Introduction

A significant number of patients experience heterogeneous sequelae following severe acute respiratory syndrome coronavirus 2 (SARS‐CoV‐2) infection, commonly known as Long COVID [1]. The World Health Organization (WHO) defines the lasting effects of coronavirus disease 2019 (COVID‐19) as a “condition that occurs in individuals with a history of probable or confirmed SARS‐CoV‐2 infection, usually 3 months from the onset of COVID‐19, with symptoms that last for at least 2 months and cannot be explained by an alternative diagnosis” [2]. The WHO consensus definition also specifies that common symptoms include fatigue, shortness of breath, and cognitive dysfunction, but also others, and they may fluctuate or relapse over time.

The reported prevalence of Long COVID varies considerably [1, 3, 4, 5, 6, 7, 8, 9, 10]. Differences in methods, population, and classification of cases (settings, severity, duration, number, and sets of symptoms) make it difficult to summarize the findings across studies [5, 7, 10]. While the highest burden is among those patients with a history of mild to moderate COVID‐19 illness, the vast majority of studies following severe COVID‐19 [3, 5, 7]. Additionally, there is the paucity of evidence on Long COVID among nonhospitalized patients from low‐ and middle‐income countries and from primary care [3, 5, 7].

Timely diagnosis of prolonged health problems following acute SARS‐CoV‐2 infection is key for preventing chronic or life‐debilitating conditions. However, there is a debate about whether the list of possible symptoms reported in people who experienced Long COVID is too long to implement an effective diagnosis [11]. Over 200 different symptoms have been identified as related to this illness, with some symptoms (e.g., fatigue, cognitive and mental dysfunction symptoms) being more frequent than others [1]. To address the high heterogeneity of findings in Long COVID research, the Core Outcome Measurement in Effectiveness Trials (COMET) Initiative defined the core outcome set (COS), which was suggested to be measured and reported in studies of Long COVID [11]. The authors noted that this minimum set of outcomes does not prohibit researchers from including other relevant outcomes.

The purpose of this study was to determine the occurrence and risk factors for persistent symptoms after mild to moderate SARS‐CoV‐2 infection in patients who were presented in primary care during the 2021 pandemic. To overcome the possible heterogeneity of self‐reported Long COVID symptoms, a cross‐sectional survey was conducted using a questionnaire with a set of eighteen validated clinical outcomes.

## 2 Methods

### 2.1 Study Design and Settings

A retrospective cross‐sectional survey was conducted in six Family Health Care Units in João Pessoa, the capital city of Paraiba State, Brazil, from May 2023 to July 2024. After completion of the validation process [12], the questionnaire with a set of 18 outcomes was applied to interview survivors of acute SARS‐CoV‐2 infection who were attended and diagnosed in ambulatory settings back in 2021.

The Family Health Care Units (Unidade de Saúde da Família ‐ USF) are ambulatory primary care facilities located throughout all territories of Brazil as a part of the National Primary Health Care Policy (Política Nacional de Atenção Básica) established by the Brazilian Ministry of Health [13]. Each USF is typically composed of family health teams made up of doctor(s), assistant nurses, and community health workers [14]. In João Pessoa, the health services network, in which USFs make a part, is distributed among five sanitary districts that cover the entire territorial extension and population of the city [15].

The process of development and application of the survey involved two main phases: (1) development and validation of the Long COVID Questionnaire; and (2) mapping of COVID‐19 cases and application of the survey in six ambulatory facilities with different territorial locations across the city. The recruitment areas varied in terms of the population, which served to allow for ethnic and sociodemographic diversity among the study participants.

### 2.2 Participants

**Figure 1:**
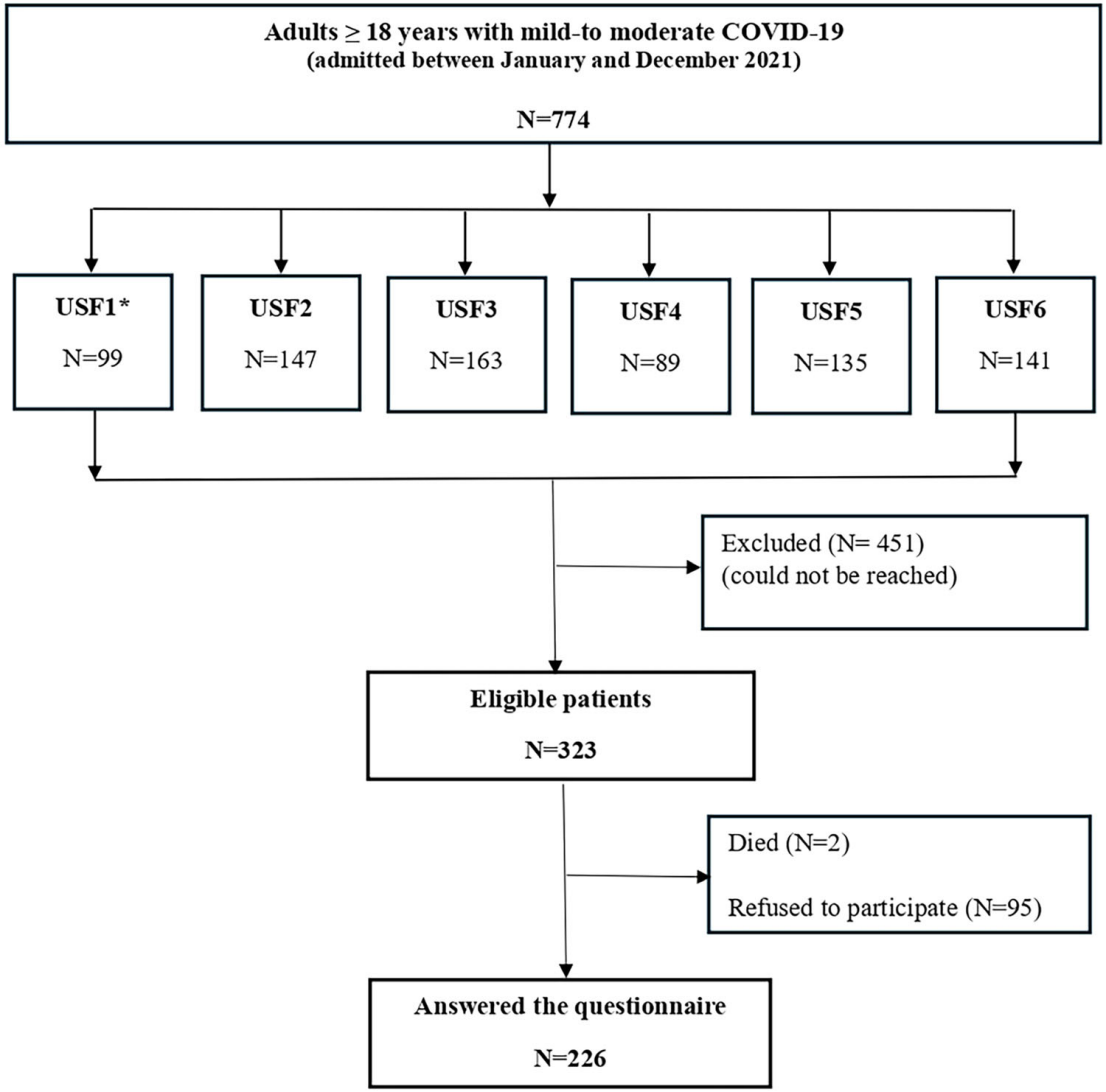
Participants selection flowchart.

This study enrolled adult patients who were admitted at USFs between January and December 2021, and who had a history of mild‐to moderate COVID‐19. The sample was composed of all patients who fulfilled the eligibility criteria: age ≥ 18 years, laboratory confirmed SARS‐CoV‐2 infection (positive nucleic acid amplification test‐NAAT), and patients who could be reached in a pre‐survey period (participants who had valid address/contact information and permanently living in Joao Pessoa). Excluded from the study were individuals who presented with an acute respiratory illness but did not test for SARS‐CoV‐2 infection or who had negative results, and those who were unable/not willing to answer the questionnaire. The list of potentially eligible individuals and their respective COVID‐19 laboratory results with contact information were obtained from USF medical records and electronic archives. Research staff reached out to potentially eligible participants via home visits or phone calls. All individuals were asked about their willingness to participate in the study and provided paper‐based informed consent. The selection procedure of the study is presented in Figure 1.

The mild‐to moderate COVID‐19 diagnosis was provided by professional doctors, who were affiliated at the USFs. The mild COVID‐19 cases were considered symptomatic patients without evidence of viral pneumonia or hypoxia. The moderate COVID‐19 included patients with clinical signs of non‐severe pneumonia, who did not require hospitalization [16].

#### 2.2.1 Ethical Considerations

The study was constructed using primary data and subject identification.

The study protocol was approved by the Ethics Committee (n 5.376.051; CAAE: 55764722.4.0000.5188), as required for conducting research involving humans.

### 2.3 Long COVID Questionnaire and Data Collection

The development of the Long COVID questionnaire was carried out via a matrix mapping concept of core clinical outcomes for Long COVID identified through international systematic reviews/meta‐analyses and cohort studies. The detailed process of its development and validation is described in a recent thesis [12]. After completion of the validation process, the set of 18 core clinical outcomes was organized into a final questionnaire.

*Long COVID outcomes*. To define Long COVID symptoms, the study followed the post‐COVID‐19 condition definition outlined by WHO [17]. We asked patients if any of the listed symptoms occurred within a period of 3 months after infection and if they lasted at four time points: less than 1 month, 1–2 months, 2–3 months, and more than 3 months. The cases that matched the Long COVID definition were considered those that occurred within 3 months after SARS‐CoV‐2 infection onset, had a duration of at least 2 months and could not be explained by any alternative diagnosis.

Eighteen Long COVID outcomes passed the validation process and were included in a questionnaire: (1) fatigue, (2) postexertional malaise, (3) sore throat, (4) muscle pain, (5) dyspnea, (6) chest pain, (7) joint pain, (8) dry cough, (9) difficulty breathing, (10) diarrhea, (11) anxiety, (12) dizziness, (13) short‐term memory loss, (14) mental confusion, (15) loss of attention, (16) difficulty thinking, (17) difficulty in executive function, and (18) difficulty in solving problems. Missing data on short‐term memory loss and difficulty thinking were denoted by the “missing” category with the corresponding variable.

*Independent variables*. Demographic characteristics (age, sex, ethnic group, level of education), presence of pre‐existing chronic conditions, and COVID‐19 vaccination status were also collected.

The age of the participants at the time of symptom onset was categorized as either < 60 and ≥ 60 years old or as a 6‐year‐old group (18–29, 30–39, 40–49, 50–59, 60–69, 70+ years old). The ethnic group was categorized as either (white, African, Asian, mixed race, Indigenous) or two‐categorical (white/nonwhite). Education level was categorized as < 12 and ≥ 12 years of schooling. Comorbidities were categorized as either present/absent (yes/no) or by their quantity (none, one, two, or more than two pre‐existing chronic conditions). The participants were asked if they had been vaccinated before SARS‐CoV‐2 infection. COVID‐19 vaccination status was categorized as none or ≥ 1 dose.

All COVID‐19‐related symptoms were categorized by their number as either dichotomous variables (< 5 and ≥ 5 symptoms) or as five categories (none, 0–4, 5–9, 10–15, and more than 15 symptoms).

The survey consisted of 47 questions and was originally written in Brazilian Portuguese (Supporting Material). All participants were interviewed in person (mostly on home visits). The interviews were provided by graduate and postgraduate medical students, who were trained and monitored by a senior. The Long COVID questionnaire was administered through KoboTool‐ a free electronic platform designed for secure data collection, management, and visualization [18]. The average time to complete a survey was estimated to be 30 min per interview.

### 2.4 Statistical Analysis

Statistical analyses were conducted via SPSS v25.0 (IBM Corp., Armonk, NY, USA).

*Baseline characteristics*. Demographics, COVID‐19 characteristics, and comorbidities are expressed as the means and standard deviations (SDs) for continuous variables (age) and as absolute values along with percentages (%) for categorical variables: sex, race/ethnicity, education, COVID‐19‐related symptom quantity, vaccination status, and pre‐existing health conditions.

Differences between the age means of the two groups, those with Long COVID and those without Long COVID, were estimated with a *t*‐test for independent samples. Between‐categorical group comparisons were performed with the chi‐squared test or Fisher's exact test, as appropriate.

*Symptoms and risk factors*. The occurrence of symptoms was expressed as a percentage by dividing the number of patients who reported experiencing a symptom by the total number of patients in the survey, with 95% confidence intervals (95% CI) estimated for proportions via the Clopper–Pearson method.

Binary logistic regression was used to estimate crude and adjusted odds ratios (ORs) with the respective 95% CIs for the factors associated with persistent symptoms. Univariate analysis was performed with each confounder before running the multivariate model. Age groups with 10‐year intervals (18–29‐*reference*, 30–39, 40–49, 50–59, 60–69, and 70+ years), sex (dichotomous, male‐*reference*/female), and the presence of comorbidities (dichotomous, not present‐*reference*/present) were included in the final model, with an average of 75% classified cases.

### 2.5 Patient and Public Involvement

Patients and the public were not involved in the design, or conduct, or reporting, or dissemination plans of this study. The results of the study will be disseminated to the public and stakeholders by press releases and publications.

## 3 Results

### 3.1 Baseline Characteristics

In total, 226 patients with confirmed SARS‐CoV‐2 infection responded to the questionnaire (Table 1). The mean age was 49.5 years (SD 15.3), and 68.1% were female. Among the participants, 73.0% were nonwhite, and 71.7% had less than 12 years of schooling. Approximately 37% had comorbidities, the most common of which were arterial hypertension (25.2%) and diabetes (11.1%). Of all patients, 98.7% received COVID‐19 vaccination at primary care.

**Table 1:** Characteristics of study participants.

Variables	All		With Long COVID		Without Long COVID		*p* value
	*N* = 226		*N* = 156		*N* = 70		
	*n*	%	*n*	%	*n*	%	
**Total**	226	100	156	69.0	70	31.0	**—**
**Sex**, Male	72	31.9	42	26.9	30	42.9	0.017
**Age in years**							
Mean, SD	49.5 (15.3)		51.0 (14.9)		46.0 (15.6)		0.021
**Symptoms within the questionnaire**							
< 5	78	34.5	28	17.9	50	71.4	< 0.001
≥ 5	148	65.5	128	82.1	20	28.6	
None	16	7.1	—	—	16	7.1	< 0.001
0–4	62	27.4	28	12.4	34	15.0	
5–9	69	30.5	57	25.2	12	5.3	
10–15	61	27.0	55	24.3	6	2.7	
> 15	18	8.0	16	7.1	2	0.9	
**COVID‐19 vaccination**							
None	3	1.3	2	1.3	1	1.4	0.928
≥ 1 dose	223	98.7	154	98.7	69	98.6	
**Age groups**							
< 60	159	70.4	107	68.6	52	74.3	0.386
≥ 60	67	29.6	49	31.4	18	25.7	
18–29	22	9.7	11	7.1	11	15.7	0.081
30–39	44	19.5	27	17.3	17	24.3	
40–49	50	22.1	40	25.6	10	14.3	
50–59	43	19.1	29	18.6	14	20.0	
60–69	40	17.7	27	17.3	13	18.6	
70+	27	11.9	22	14.1	5	7.1	
**Race/Ethnicity**							
White	61	27.0	42	26.9	19	27.1	0.973
Non‐White	165	73.0	114	73.1	51	72.9	
**Education level**							
< 12 years	162	71.7	107	68.6	55	78.6	0.113
≥ 12 years	61	27.0	47	30.1	14	20.0	
ND	3	1.3	2	1.3	1	1.4	
**Comorbidities**							
None	143	63.3	94	60.3	49	70.0	0.483
Any	83	36.7	62	39.7	21	30.0	
Arterial hypertension	57	25.2	43	27.6	14	20.0	0.226
Diabetes mellitus	25	11.1	17	10.9	8	11.4	0.906
Musculoskeletal diseases	11	4.9	9	5.8	2	2.9	0.347
Respiratory diseases	4	1.8	4	2.6	—	—	0.176
Cardiac diseases	3	1.3	1	0.6	2	2.9	0.177
Other^a^	18	8.0	16	10.3	2	2.9	0.026

Abbreviations: ND, information not declared; SD, standard deviation.

^a^ Category “other comorbidities” includes anemia, asthma, allergy, chronic sinusitis, thyroid diseases, eye conditions.

#### 3.1.1 Characteristics of Participants With Long COVID

Seven percent (16/226) of all patients were symptom‐ free 3 months after SARS‐CoV‐2 infection onset. Among all the participants, 69.0% (156/226) met the definition of Long COVID and had at least one prolonged symptom after COVID‐19 illness. Of patients with Long COVID, 73.1% (114/156) were female, 68.6% (107/156) were younger than 60 years, 73.1% (114/156) were nonwhite, and 39.7% (62/156) had at least one pre‐existing health condition. Adults of age 70+ years had the highest proportion (68.2%) of comorbidities compared to other age groups (Supporting Information S1: Table S1). Compared with those without Long COVID, patients with Long COVID were older (mean 51 years old vs. 46 years old, *p* = 0.02), were more likely to be female (41.9% vs. 26.9%, *p* = 0.017), and did not differ in terms of other demographic characteristics (Table 1).

Eighty‐two percent of patients (128/156) with Long COVID reported five or more symptoms (Table 1). Among them, 35.3% reported between 10 and 15 symptoms, and 10.3% reported more than 15 symptoms. The frequency of COVID‐19‐related symptoms ≥ 5 was significantly greater in patients with Long COVID than in those who did not present prolonged symptoms (82.1% vs. 28.6%, *p* < 0.001).

### 3.2 Symptoms

**Figure 2:**
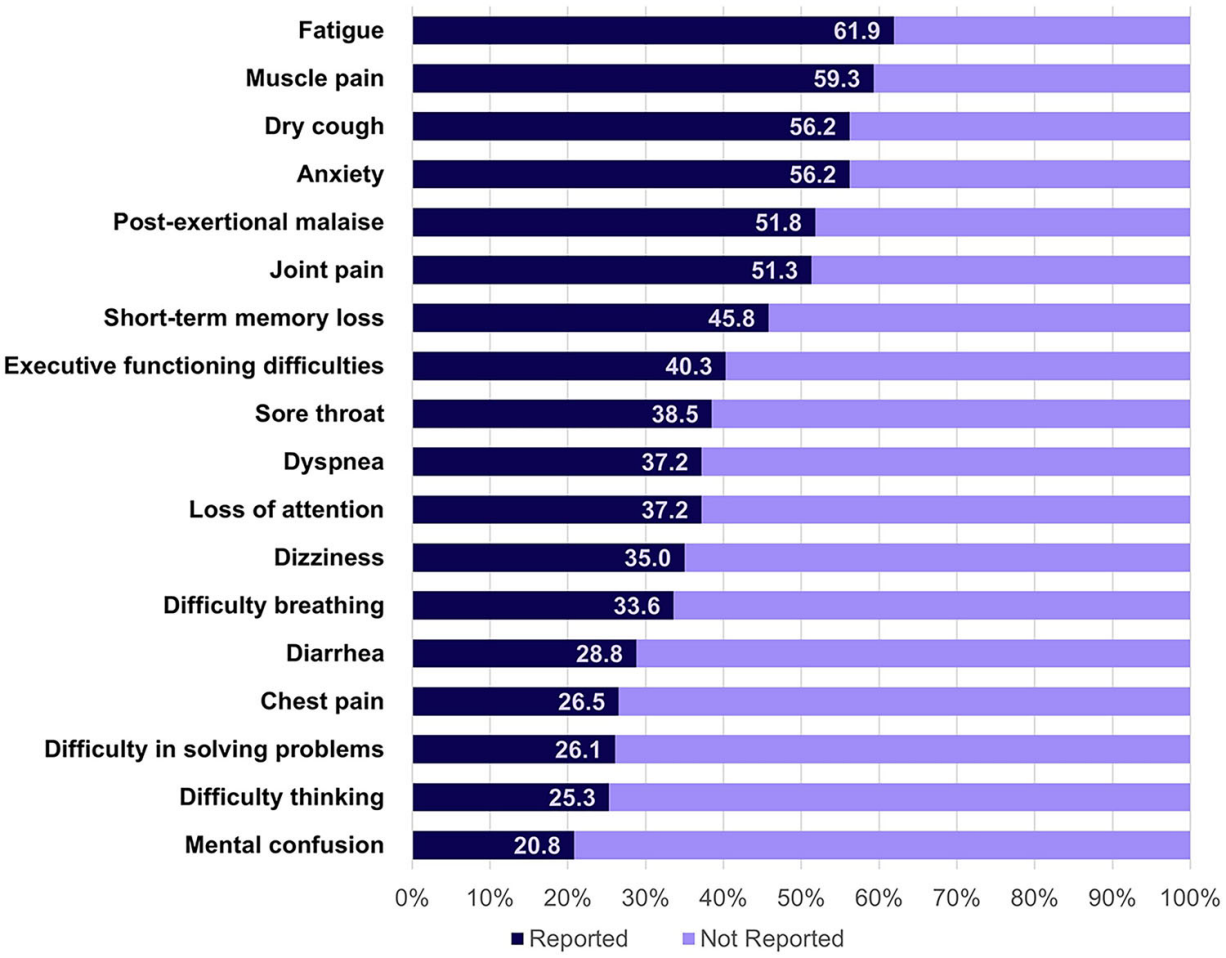
Frequency of symptoms reported by patients after mild‐to moderate COVID‐19.

Among the 18 outcomes included in the questionnaire, the most frequently reported by patients were fatigue (61.9%), muscle pain (59.3%), dry cough (56.2%), anxiety (56.2%), and short‐term memory loss (45.8%) (Figure 2). They were followed by executive functioning difficulties (40.3%), sore throat (38.5%), dyspnea (37.2%), loss of attention (37.2%), dizziness (35.0%), difficulty breathing (33.6%), diarrhea (28.8%), chest pain (26.5%), difficulty thinking (25.3%), and mental confusion (20.8%).

Among all the recorded symptoms, 16 (16/18) were significantly different in terms of occurrence per subgroup (with Long COVID vs. without Long COVID), except for sore throat and diarrhea (Table 2). The greatest differences were found for short‐term memory loss (62.6% vs. 8.6%, *p* < 0.0001), loss of attention (50.0% vs. 8.6%, *p* < 0.0001), anxiety (71.8% vs. 21.4%, *p* < 0.0001), joint pain (65.4% vs. 20.0%, *p* < 0.0001), difficulty thinking (33.5% vs. 7.1%, *p* < 0.0001), dizziness (45.5% vs. 11.4%, *p* < 0.0001), difficulty breathing (42.3% vs 14.3%, *p* < 0.0001), and postexertional malaise (62.8% vs. 27.1%, *p* < 0.0001).

**Table 2:** Frequency of symptoms comparing patients with versus without long COVID.

Symptoms	With long COVID *N* = 156			Without long COVID *N* = 70			*p* value
	*n*	%	95% CI	*n*	%	95% CI	
Fatigue	112	0.718	0.640–0.787	28	0.4	0.285–0.524	< 0.0001
Anxiety	112	0.718	0.640–0.787	15	0.214	0.125–0.329	< 0.0001
Muscle pain	105	0.673	0.593–0.746	29	0.414	0.298–0.538	0.00026
Joint pain	102	0.654	0.574–0.728	14	0.2	0.114–0.313	< 0.0001
Dry cough	101	0.647	0.567–0.722	26	0.371	0.259–0.495	< 0.0001
Postexertional malaise	98	0.628	0.547–0.704	19	0.271	0.172–0.391	< 0.0001
Short‐term memory loss^a^	97	0.626	0.545–0.702	6	0.086	0.032–0.177	< 0.0001
Loss of attention	78	0.5	0.419–0.581	6	0.086	0.032–0.177	< 0.0001
Executive functioning difficulties	76	0.487	0.406–0.568	15	0.214	0.125–0.329	< 0.0001
Dizziness	71	0.455	0.375–0.537	8	0.114	0.051–0.213	< 0.0001
Dyspnea	70	0.449	0.369–0.530	14	0.2	0.114–0.313	0.00034
Difficulty breathing	66	0.423	0.344–0.505	10	0.143	0.071–0.247	< 0.0001
Sore throat	65	0.417	0.338–0.498	22	0.314	0.209–0.436	0.144
Difficulty thinking^a^	52	0.335	0.262–0.416	5	0.071	0.024–0.159	< 0.0001
Diarrhea	51	0.327	0.254–0.407	14	0.2	0.114–0.313	0.051
Difficulty in solving problems	50	0.321	0.248–0.400	9	0.129	0.061–0.230	< 0.0001
Chest pain	49	0.314	0.242–0.393	11	0.157	0.081–0.264	0.014
Mental confusion	43	0.276	0.207–0.353	4	0.057	0.016–0.140	< 0.0001

Abbreviation: CI, confidence interval.

^a^ One missing value.

There is a difference in the prevalence of persistent symptoms regarding age groups (Supporting Information S1: Table S2). Fatigue was frequently reported by adults of all ages, with the highest proportion (60%) among adults aged 40–49 years. Other commonly reported symptoms were: anxiety (36.4%) in adults aged 18–29 years (36.4%); anxiety (38.6%) and short‐term memory loss (38.6%) in adults aged 30–39 years (43.2%); postexertional malaise (60%) in adults aged 40–49 years (60%); joint pain in adults aged 50–59 years (58.1%); muscle pain in adults aged 50–59 years (52.5%); dry cough (59.3%) and postexertional malaise (55.6%) in adults aged 70+ years.

**Figure 3:**
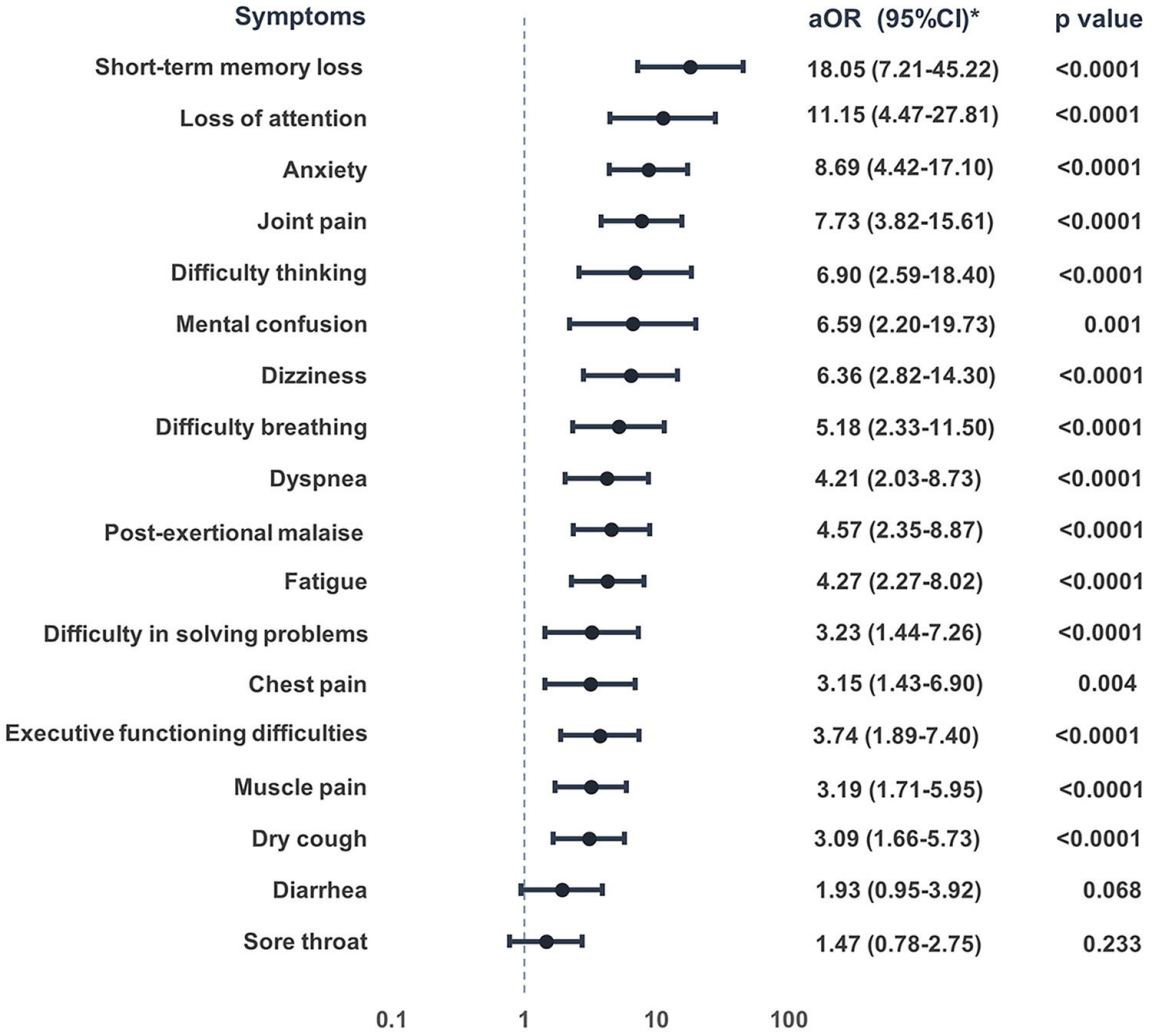
Adjusted odds ratios for persistent symptoms after mild‐to moderate COVID‐19. Odds ratio (OR), confidence interval (CI), *‐adjusted for sex, age, and comorbidities.

The occurrence of persistent symptoms after COVID‐19 was significantly associated with a total of 16 outcomes after adjustment for sex, age, and the presence of comorbidities (Figure 3). The symptoms with the largest aOR were short‐term memory loss (aOR: 18.05, 95% CI: 7.20–45.22), loss of attention (aOR: 11.15, 95% CI: 4.47–27.81), anxiety (aOR: 8.69, 95% CI: 4.42–17.10), joint pain (aOR: 7.73, 95% CI: 3.82–15.61), difficulty thinking (aOR: 6.90, 95% CI: 2.59–18.40), dizziness (aOR: 6.36, 95% CI: 2.82–14.34), difficulty breathing (aOR: 5.18, 95% CI: 2.33–11.54), and postexertional malaise (aOR: 4.57, 95% CI: 2.35–8.87).

### 3.3 Risk Factors

In the multivariate logistic regression analyses of patients infected with SARS‐CoV‐2, a significant association was found between persistent symptoms and female sex, age 40–49 years, age 70+ years, and the presence of ≥ 5 symptoms (Table 3). Compared with men, women were at increased risk (aOR: 1.91, 95% CI: 1.03–3.53, *p* = 0.032). Both the 40–49 years old and 70+ years old age groups were associated with a higher risk of reporting persistent symptoms in the univariate analysis. After adjustment for covariates, those aged 40‐49 years presented an approximately 3.5‐fold increased risk (aOR: 3.45, 95% CI: 1.14–10.51, *p* = 0.029), and those aged 70+ years presented a fourfold increased risk (aOR: 4.0, 95% CI: 1.03–15.50, *p* = 0.045) compared with people aged 18–29 years. There was a statistically significant association between reported Long COVID and the number of COVID‐19‐related symptoms. Patients with COVID‐19 who initially reported ≥ 5 symptoms had a significantly increased risk for Long COVID, compared with those who reported < 5 symptoms (aOR: 15.82, 95% CI: 7.33–34.15, *p* < 0.0001).

**Table 3:** Risk factors associated with long COVID in patients presented at primary care.

Variables	With long COVID	Without long COVID				
	*N* = 156	*N* = 70	Unadjusted OR		Adjusted OR^b^	
	*n*	*n*	OR (95% CI)	*p* value	aOR (95% CI)	*p* value
**Symptoms**						
< 5	28	50	**Ref.**		**Ref.**	
≥ 5	128	20	11.43 (5.90–22.12)	< 0.0001	15.82 (7.33–34.15)	**< 0.0001**
**Sex**						
Male	42	30	**Ref.**		**Ref.**	
Female	114	40	2.04 (1.13–3.68)	0.018	1.91 (1.03–3.53)	**0.032**
**Age**						
< 60	107	52	**Ref.**		**Ref.**	
≥ 60	49	18	1.32 (0.70–2.49)	0.387	1.22 (0.62–2.41)	0.564
18–29	11	11	**Ref.**		**Ref.**	
30–39	27	17	1.59 (0.57–4.46)	0.38	1.56 (0.55–4.43)	0.408
40–49	40	10	4.0 (1.35–11.84)	0.012	3.45 (1.14– 10.51)	**0.029**
50–59	29	14	2.07 (0.72–5.93)	0.175	1.75 (0.59–5.19)	0.312
60–69	27	13	2.01 (0.72–6.03)	0.179	1.81 (0.59–5.53)	0.298
70+	22	5	4.4 (1.22–15.84)	0.023	4.0 (1.01–15.51)	**0.045**
**Ethnicity**						
White	42	19	**Ref.**		**Ref.**	
Nonwhite	114	51	1.01 (0.54–1.91)	0.973	1.07 (0.55–2.08)	0.843
**Education** a						
< 12^c^	107	55	**Ref.**		**Ref.**	
≥ 12	47	14	1.73 (0.88–3.41)	0.116	2.04 (0.99–4.21)	0.053
**Comorbidities**						
None	94	49	**Ref.**		**Ref.**	
Any	62	21	1.54 (0.84–2.81)	0.161	1.13 (0.57–2.23)	0.723
AH	43	14	1.52 (0.77–3.01)	0.228	1.21 (0.38–3.88)	0.738
Diabetes	17	8	0.95 (0.39–2.31)	0.906	0.56 (0.19–1.68)	0.303

*Note:* Bold values indicate statistically significant results.

Abbreviations: AH, arterial hypertension; aOR, adjusted odds ratio; CI, confidence interval; OR, odds ratio.

^a^ Two missing values.

^b^ Adjusted with sex, age and comorbidities.

^c^ Years of schooling.

## 4 Discussion

The occurrence and risk factors for persistent symptoms after mild to moderate COVID‐19 were assessed in 226 patients who attended in Brazilian public primary care facilities during the 2021 pandemic. A standardized questionnaire with a set of 18 validated clinical outcomes was applied to describe Long COVID. More than two‐thirds of respondents reported at least one persistent symptom 3 months or more after the acute phase of SARS‐CoV‐2 infection. Only a small number of patients were completely symptom‐free or experienced short‐term effects.

In the present study, a high proportion of adults who are predominantly of working age had continuing health problems after non‐severe COVID‐19 illness, which is particularly striking. The prevalence of Long COVID varies significantly across the studies, with far more available data that followed posthospital periods [3, 10, 19, 20]. A meta‐analysis on the global prevalence of Long COVID among a total of 1.68 million COVID‐positive patients reported a higher pooled prevalence in hospitalized cohorts than in nonhospitalized cohorts (0.54 vs. 0.34, respectively) [19]. In another meta‐analysis, 45% of COVID‐19 hospitalized and nonhospitalized survivors experienced at least one long‐haul symptom, regardless of hospitalization status [7]. Both meta‐analyses described the geographical asymmetry of available studies with majority of data from Europe [7, 19].

A few previous studies from Brazil explored persistent symptoms among COVID‐19 survivors [21, 22, 23, 24, 25, 26, 27, 28, 29]. The prevalence of Long COVID in these studies depends on the severity of initial acute infection, settings, duration of symptoms, and methodology of assessment. The studies based on previously hospitalized [21, 23, 24, 26] or mixed cases [25, 28, 29] reported long‐lasting symptoms in 41.5%–100% of patients. One previous study of 140 nonhospitalized patients reported Long COVID in 44.3% of adults 1 year after acute infection [27], which is lower compared to the estimated prevalence in our study. However, this study used online questionnaires, which may misrepresent those patients who were not able to fill in the online forms. Additionally, the study was conducted in the South region of Brazil [27], which has higher socioeconomic indexes as compared to the North‐East region, where our study was conducted; hence, it may impact differently the people's health outcomes [30].

Among the 18 predefined outcomes in the present study, 16 were significantly associated with Long COVID. Neurological and neuropsychiatric symptoms stood out as the more commonly reported persistent symptoms among all the participants in our study. Fatigue was the most prevalent symptom, which was identified in more than two‐third of patients with Long COVID. Muscle pain, postexertional malaise, anxiety, and short‐term memory loss were also highly common symptoms and were identified in more than half of adults in this group. Moreover, along with memory issues, about one‐third of patients with long‐haul COVID experienced other cognitive signs such as difficulty thinking, loss of attention and difficulty in solving problems.

Previous studies have identified a wide range of neurological and neuropsychiatric impairments during and after the acute phase of COVID‐19 illness [5, 6, 7, 25, 31, 32, 33, 34], with few studies in the nonhospitalized cohorts [25, 32]. A meta‐analysis of over 10 000 individuals from 18 studies describes neurological and neuropsychiatric symptoms both highly common 3 months after an acute COVID‐19 infection [31]. The cognitive impairments in this analysis were found to be more prevalent in nonhospitalized patients as compared with previously hospitalized patients (51% vs. 32%). Additionally, fatigue and cognitive symptoms showed a strong tendency to progress over time [31, 33]. The results of a large cross‐sectional cognitive performance study of 84 285 survivors of COVID‐19 describe significant cognitive deficits in these patients versus controls [32]. Cognitive symptoms remained present even in those who had milder COVID‐19 cases and those who no longer had other residual symptoms, and could not be explained by differences in age, education, and other sociodemographic characteristics [32]. A wide range of common neurologic and neuropsychiatric symptoms in our study along with other symptoms experienced by patients with Long COVID after non‐severe infection may point to larger health issues in the future. Our findings highlight the importance of long‐term assistance in primary care not only for previously hospitalized patients who required rehabilitation, but also for those who continue experiencing health problems after milder illness.

In our study, female sex, age 40–49 years and 70+ years, and five or more initial symptoms were associated with Long COVID. A higher number of COVID‐19 related symptoms was the strongest predictor of Long COVID in our cohort. Female sex [6, 7, 8, 9, 10, 19, 20, 21, 23, 25, 29, 33, 35] and multiple symptoms [7, 8, 9, 33] were consistently identified as significant risk factors for Long COVID, regarding hospitalization status. However, there are conflicting findings on the association between Long COVID and certain age. Studies have identified different age groups as those that are potentially at risk for developing post‐COVID conditions, possibly due to differences in observed populations, age stratification and severity of infection. Some studies reported increased risk with older ages [20, 23, 24], while other studies reported increased risk among younger and middle‐aged adults [9, 20, 33, 34, 35].

Older adults have a greater risk of morbidity and mortality from COVID‐19 than younger adults [36] and are also at risk of Long COVID [37]. Adults aged 70+ years had the highest proportion of comorbidities in our study, including diabetes and hypertension, compared to other age groups, which may contribute to poorer outcomes. Furthermore, the evidence suggests that SARS‐CoV‐2 infection can exacerbate existing chronic conditions [36, 37]. Additionally, the general decline in physiological functions, lower capacity of cell regeneration, and less robust immune responses in elderly may render them more susceptible to tissue‐damaging immunity and chronic inflammation [38]. Group of adults aged 40–49 years comprises more women and has the highest proportion of symptoms ≥ 5 compared to other age groups, which could double the risk. However, the exact pathophysiological mechanism of Long COVID in middle‐aged adults is not fully understood.

Although substantial progress has been made in defining mechanisms of Long COVID development, some questions remain uncovered due to complex human biology and pathophysiology [39]. Several mechanisms have been described, including postacute virus persistence [40, 41], postacute inflammation [42], immune dysregulation and autoimmunity [43, 44], reactivation of other pathogens [45], disruption of the gut microbiome [46], mitochondrial dysfunction [47], and microvascular blood clotting with endothelial dysfunction [48]. Additionally, microglial reactivity with consequent neural dysregulation was found to play a central role in neurocognitive manifestations of Long COVID [49].

A strength of our study was the assessment of Long COVID using a standardized questionnaire with a set of predefined validated outcomes, which could be useful for assessing Long COVID in primary care practice, especially in resource‐limited settings. Symptoms reported by patients were narrowed to avoid excessive heterogeneity. The study was conducted in several public ambulatory settings, which varied in terms of the resident population. Additionally, a face‐to‐face interview permitted us to better represent the live experiences of COVID‐19 survivors.

This study has several limitations, and any generalization of the results should be considered cautiously. A key limitation of this study is relatively small sample size. A larger cohort would enhance the statistical power of the findings. Another limitation is the retrospective nature of a survey, which may introduce potential recall biases. Although most adults in this study were vaccinated within the universal Brazilian COVID‐19 vaccination program, we could not be able to track if they were vaccinated before or after infection. More women responded to our questionnaire; thus, it may influence the results. Furthermore, the testing capacity for SARS‐CoV‐2 infection during the 2021 pandemic was very limited within primary care in Brazil; some individuals may not be tested, or all positive test results may not be routinely coded.

## 5 Conclusion

In the present study, a high proportion of patients had prolonged symptoms after non‐severe SARS‐CoV‐2 infection. The occurrence of Long COVID symptoms were associated with a greater number of symptoms, female sex, age 40‐49 years and age 70 years and above. This study supports the need for assessing Long COVID clinical outcomes and risk factors in primary care using routinely recorded clinical outcomes.

## Author Contributions

Assel Muratovna Shigayeva Ferreira, Flávia Emília Leite de Lima Ferreira, Leandro Pernambuco, Caio César Ferreira Alverga, André Luís Bonifácio de Carvalho, Gabriel Rodrigues Martins de Freitas and João Agnaldo do Nascimento were responsible for conceptualization, study design, analytic approach and data curation. Assel Muratovna Shigayeva Ferreira performed statistical analysis and wrote the original manuscript draft, which was supervised by Flávia Emília Leite de Lima Ferreira and Leandro Pernambuco. Assel Muratovna Shigayeva Ferreira, Caio César Ferreira Alverga, Cleidilaine Ramos de Oliveira, Ruth Maria Mendonça Anacleto, João Aurílio Cardoso de Moraes, Izabele da Silva Rocha, Lucas Tomaz da Silva, Beatriz Carolinny Pereira da Silva Alves and José Ricardo Araujo Cardoso were responsible for data collection. All authors contributed to critically revising and approved the final version of the manuscript for publication. The corresponding author (Assel Muratovna Shigayeva Ferreira) attests that all listed authors meet authorship criteria and that no others meeting the criteria have been omitted.

## Conflicts of Interest

The authors declare no conflicts of interest.

## Supporting information


**Appendix Table 1:** Number and percentage of patients with COVID‐19 stratified by age groups. **Appendix Table 2:** Frequencies of COVID 19‐related symptoms stratified by age groups.

## Data Availability

The data that support the findings of this study are available on request from the corresponding author. The data are not publicly available due to privacy or ethical restrictions.
