# Supplementary material for: Symptoms and Risk Factors for Long COVID: A Cross‐Sectional Study in Primary Care
Source: J Med Virol. 2025 Aug 29;97(9):e70579. doi: 10.1002/jmv.70579 (PMC12396170; doi:10.1002/jmv.70579)
Supplement: Supplementary file 1 — Appendix Table 1: Number and percentage of patients with COVID‐19 stratified by age groups. Appendix Table 2: Frequencies of COVID 19‐related symptoms stratified by age groups. [file JMV-97-e70579-s001.docx]

Manuscript ID JMV-25-24586

**Symptoms and risk factors for Long COVID: a cross-sectional study in primary care.**

**Appendix-** Additional information on a retrospective cross-sectional study of patients with Long COVID

**Appendix Table 1**- Number and percentage of patients with COVID-19 stratified by age groups

| **Variables** | **All** | | **With Long COVID** | | **Without Long COVID** | | **p-value*** |
| --- | --- | --- | --- | --- | --- | --- | --- |
|  | **(N=226)** | | **(N=156)** | | **(N=70)** | |  |
|  | **n** | **%** | **n** | **%** | **n** | **%** |  |
| **18-29** | 22/226 | 9.7 | 11/22 | 50.0 | 11/22 | 50.0 |  |
| **Sex** |  |  |  |  |  |  |  |
| male | 10/22 | 45.5 | 4/11 | 36.4 | 6/11 | 54.5 | 0.670 |
| female | 12/22 | 54.5 | 7/11 | 63.6 | 5/11 | 45.5 |  |
| **Comorbidities** |  |  |  |  |  |  |  |
| yes | 2/22 | 9.1 | 1/11 | 9.1 | 1/11 | 9.1 | 1.0 |
| no | 20/22 | 90.9 | 10/11 | 90.9 | 10/11 | 90.9 |  |
| **Symptoms** |  |  |  |  |  |  |  |
| <5 | 6/22 | 27.3 | 2/11 | 18.2 | 4/11 | 36.4 | 0.635 |
| ≥5 | 16/22 | 72.7 | 9/11 | 81.8 | 7/11 | 63.6 |  |
| **30-39** | 44/226 | 19.5 | 27/44 | 61.4 | 17/44 | 38.6 |  |
| **Sex** |  |  |  |  |  |  |  |
| male | 18/44 | 40.9 | 11/27 | 40.7 | 7/17 | 41.2 | 1.0 |
| female | 26/44 | 59.1 | 16/27 | 59.3 | 10/17 | 58.8 |  |
| **Comorbidities** |  |  |  |  |  |  |  |
| yes | 5/44 | 11.4 | 5/27 | 18.5 | 0/17 | 0.0 | 0.139 |
| no | 39/44 | 88.6 | 22/27 | 81.5 | 17/17 | 100.0 |  |
| **Symptoms** |  |  |  |  |  |  |  |
| <5 | 22/44 | 50.0 | 7/27 | 25.9 | 15/17 | 88.2 | <0.001 |
| ≥5 | 22/44 | 50.0 | 20/27 | 74.1 | 2/17 | 11.8 |  |
| **40-49** | 50/226 | 22.1 | 40/50 | 80.0 | 10/50 | 20.0 |  |
| **Sex** |  |  |  |  |  |  |  |
| male | 12/50 | 24.0 | 7/40 | 17.5 | 5/10 | 50.0 | 0.046 |
| female | 38/50 | 36.0 | 33/40 | 82.5 | 5/10 | 50.0 |  |
| **Comorbidities** |  |  |  |  |  |  |  |
| yes | 18/50 | 36.0 | 14/40 | 35.0 | 4/10 | 40.0 | 1.0 |
| no | 32/50 | 64.0 | 26/40 | 65.0 | 6/10 | 60.0 |  |
| **Symptoms** |  |  |  |  |  |  |  |
| <5 | 11/50 | 22.0 | 4/40 | 10.0 | 7/10 | 70.0 | <0.001 |
| ≥5 | 39/50 | 78.0 | 36/40 | 90.0 | 3/10 | 30.0 |  |
| **50-59** | 43/226 | 19.0 | 29/43 | 67.4 | 14/43 | 32.6 |  |
| **Sex** |  |  |  |  |  |  |  |
| male | 10/43 | 23.3 | 4/29 | 13.8 | 6/14 | 42.9 | 0.055 |
| female | 33/43 | 76.7 | 25/29 | 86.2 | 8/14 | 57.1 |  |
| **Comorbidities** |  |  |  |  |  |  |  |
| yes | 17/43 | 39.5 | 13/29 | 44.8 | 4/14 | 28.6 | 0.343 |
| no | 26/43 | 60.5 | 16/29 | 55.2 | 10/14 | 71.4 |  |
| **Symptoms** |  |  |  |  |  |  |  |
| <5 | 17/43 | 39.5 | 5/29 | 17.2 | 12/14 | 85.7 | <0.001 |
| ≥5 | 26/43 | 21.8 | 24/29 | 82.8 | 2/14 | 14.3 |  |
| **60-69** | 40/226 | 17.7 | 27/40 | 67.5 | 13/40 | 32.5 |  |
| **Sex** |  |  |  |  |  |  |  |
| male | 12/40 | 30.0 | 6/27 | 22.2 | 6/13 | 46.2 | 0.154 |
| female | 28/40 | 70.0 | 21/27 | 77.8 | 7/13 | 53.8 |  |
| **Comorbidities** |  |  |  |  |  |  |  |
| yes | 21/40 | 52.5 | 14/27 | 51.9 | 7/13 | 53.8 | 1.0 |
| no | 19/40 | 47.5 | 13/27 | 48.1 | 6/13 | 46.2 |  |
| **Symptoms** |  |  |  |  |  |  |  |
| <5 | 11/40 | 27.5 | 3/40 | 11.1 | 8/13 | 61.5 | 0.002 |
| ≥5 | 29/40 | 72.5 | 24/29 | 88.9 | 5/13 | 38.5 |  |
| **70+** | 27/226 | 11.9 | 22/27 | 81.5 | 5/27 | 18.5 |  |
| **Sex** |  |  |  |  |  |  |  |
| male | 10/27 | 37.0 | 10/22 | 45.5 | 0/5 | 0.0 | 0.124 |
| female | 17/27 | 63.0 | 12/22 | 54.5 | 5/5 | 100.0 |  |
| **Comorbidities** |  |  |  |  |  |  |  |
| yes | 20/27 | 74.1 | 15/22 | 68.2 | 5/5 | 100.0 | 0.283 |
| no | 7/27 | 25.9 | 7/22 | 31.8 | 0/5 | 0.0 |  |
| **Symptoms** |  |  |  |  |  |  |  |
| <5 | 11/27 | 40.7 | 7/22 | 31.8 | 4/5 | 80.0 | 0.125 |
| ≥5 | 16/27 | 59.3 | 15/22 | 68.2 | 1/5 | 20.0 |  |
| *-Fisher’s exact test; statistical significance p<0.05 | | | | | | | |
|  |  |  |  |  |  |  |  |

**Appendix Table 2**-Frequencies of COVID 19-related symptoms stratified by age groups

| **Symptoms** | **18-29 (n=22)** | | **30-39 (n=44)** | | **40-49 (n=50)** | | **50-59 (n=43)** | | **60-69 (n=40)** | | **70+ (n=27)** | |
| --- | --- | --- | --- | --- | --- | --- | --- | --- | --- | --- | --- | --- |
|  | **LC** | **No LC** | **LC** | **No LC** | **LC** | **No LC** | **LC** | **No LC** | **LC** | **No LC** | **LC** | **No LC** |
|  | **n (%)** | **n (%)** | **n (%)** | **n (%)** | **n (%)** | **n (%)** | **n (%)** | **n (%)** | **n (%)** | **n (%)** | **n (%)** | **n (%)** |
| **FA** | 9 (40.9) | 6 (27.3) | 19 (43.2) | 6 (13.6) | 30 (60.0) | 3 (6.0) | 22 (51.2) | 4 (9.3) | 18 (45.0) | 8 (20.0) | 14 (51.9) | 1 (3.7) |
| **PEM** | 6 (27.3) | 5 (22.7) | 11 (25.0) | 3 (6.8) | 30 (60.0) | 2 (4.0) | 21 (48.8) | 2 (4.7) | 15 (37.5) | 5 (12.5) | 15 (55.6) | 2 (7.4) |
| **ST** | 1 (4.5) | 7 (31.8) | 9 (20.5) | 2 (4.5) | 22 (44.0) | 3 (6.0) | 14 (32.6) | 4 (9.3) | 12 (30.0) | 5 (12.5) | 7 (25.9) | 1 (3.7) |
| **MP** | 7 (31.8) | 7 (31.8) | 13 (29.5) | 7 (15.9) | 29 (58.0) | 3 (6.0) | 22 (51.2) | 5 (11.6) | 21 (52.5) | 6 (15.0) | 13 (48.1) | 1 (3.7) |
| **DP** | 6 (27.3) | 5 (22.7) | 13 (29.5) | 2 (4.5) | 20 (40.0) | 1 (2.0) | 8 (18.6) | 1 (2.3) | 15 (37.5) | 5 (12.5) | 8 (29.6) | 0 (0.0) |
| **CP** | 6 (27.3) | 4 (18.2) | 11 (25.0) | 2 (4.5) | 12 (24.0) | 0 (0.0) | 10 (23.3) | 2 (4.7) | 4 (10.0) | 2 (5.0) | 6 (22.2) | 1 (3.7) |
| **JP** | 5 (22.7) | 4 (18.2) | 16 (36.4) | 2 (4.5) | 23 (46.0) | 1 (2.0) | 25 (58.1) | 2 (4.7) | 18 (45.0) | 3 (7.5) | 15 (55.6) | 2 (7.4) |
| **DC** | 4 (18.2) | 4 (18.2) | 17 (38.6) | 8 (18.2) | 27 (54.0) | 4 (8.0) | 16 (37.2) | 3 (7.0) | 21 (52.5) | 5 (12.5) | 16 (59.3) | 2 (7.4) |
| **DB** | 7 (31.8) | 4 (18.2) | 11 (25.0) | 1 (2.3) | 19 (38.0) | 1 (2.0) | 9 (20.9) | 0 (0.0) | 11 (27.5) | 4 (10.0) | 9 (33.3) | 0 (0.0) |
| **DR** | 2 (9.1) | 3 (13.6) | 7 (15.9) | 4 (9.1) | 13 (26.0) | 1 (2.0) | 7 (16.3) | 2 (4.7) | 14 (35.0) | 4 (10.0) | 8 (29.6) | 0 (0.0) |
| **ANX** | 8 (36.4) | 3 (13.6) | 17 (38.6) | 2 (4.5) | 28 (56.0) | 3 (6.0) | 24 (55.8) | 2 (4.7) | 21 (52.5) | 5 (12.5) | 14 (51.9) | 0 (0.0) |
| **DIZ** | 4 (18.2) | 1 (4.5) | 8 (18.2) | 1 (2.3) | 19 (38.0) | 3 (6.0) | 13 (30.2) | 1 (2.3) | 15 (37.5) | 2 (5.0) | 12 (44.4) | 0 (0.0) |
| **STMLᵃ** | 6 (27.3) | 2 (9.1) | 17 (38.6) | 0 (0.0) | 27 (54.0) | 0 (0.0) | 21 (48.8) | 1 (2.3) | 15 (37.5) | 3 (7.5) | 11 (40.7) | 0 (0.0) |
| **MC** | 4 (18.2) | 1 (4.5) | 7 (15.9) | 1 (2.3) | 10 (20.0) | 0 (0.0) | 8 (18.6) | 0 (0.0) | 9 (22.5) | 2 (5.0) | 5 (18.5) | 0 (0.0) |
| **LA** | 6 (27.3) | 1 (4.5) | 14 (31.8) | 1 (2.3) | 22 (44.0) | 0 (0.0) | 15 (34.9) | 1 (2.3) | 13 (32.5) | 3 (7.5) | 8 (29.6) | 0 (0.0) |
| **DT ᵃ** | 6 (27.3) | 0 (0.0) | 11 (25.0) | 0 (0.0) | 15 (30.0) | 1 (2.0) | 10 (23.3) | 2 (4.7) | 5 (12.5) | 2 (5.0) | 5 (18.5) | 0 (0.0) |
| **EFD** | 4 (18.2) | 3 (13.6) | 12 (27.3) | 1 (2.3) | 18 (36.0) | 2 (4.0) | 17 (39.5) | 4 (9.3) | 5 (12.5) | 2 (5.0) | 8 (29.6) | 1 (3.7) |
| **DSP** | 3 (13.6) | 1 (4.5) | 7 (15.9) | 1 (2.3) | 15 (30.0) | 3 (6.0) | 8 (18.6) | 1 (2.3) | 12 (30.0) | 3 (7.5) | 5 (18.5) | 0 (0.0) |
| **LC-Long COVID**, FA-fatique, PEM- post-exertional malaise, ST-sore throat, MP-muscle pain, DP-dyspnea, CP-chest pain, JP-joint pain, DC-dry cough, DB-difficulty breathing,  DR-diarrhea, ANX- anxiety, DIZ-dizziness, STML-short-term memory loss, MC-mental confusion, LA- loss of attention, DT-difficulty thinking, EFD- executive functioning difficulties, DSP-difficulty in solving problems.  **ᵃ** - one missing value. | | | | | | | | | | | | |

**Appendix -** Long COVID Questionnaire *(English version* ***^a^****)*

|  | **Items** | **Response options** |
| --- | --- | --- |
| **GENERAL INFORMATION.**  *Verify the complete name and date of birth of the participant via identification document (CPF-Cadastro de Pessoas Físicas or RG-Registro Geral).* | | |
|  | Date of interview | DD/MM/YY |
|  | Location | Neighborhood/USF* of reference |
|  | Name of the interviewer | Code name |
| 01 | What is your name? | Full name (given name, middle name, family name) |
| 02 | What is your phone number? | Code of the state, telephone number |
| 03 | What is your address? | Complete address |
| 04 | What is your age? | Age/Date of birth |
| 05 | What is your gender? | - Male |
|  |  | - Female |
|  | If other, please specify | - Other |
|  |  | - Prefer not to answer |
| 06 | What is your level of education? | - Primary school incomplete |
|  |  | - Primary school complete |
|  |  | - Secondary school incomplete |
|  |  | - Secondary school complete |
|  |  | - Technical/professional course incomplete |
|  |  | - Technical/professional course complete |
|  |  | - Higher education incomplete |
|  |  | - Higher education complete |
|  |  | - Post-graduation course incomplete |
|  |  | - Post-graduation course complete |
|  |  | - NS |
| 07 | What is your ethnic origin? | - White |
|  |  | - Black/African |
|  |  | - Asian/Oriental |
|  |  | - Mixed |
|  |  | - Indigenous |
|  |  | - NS |
| 08 | Did you have any chronic diseases before COVID-19 illness? | - Yes - No   *If Yes, can you specify disease(s)?*  *Open question* |
| 09 | Have you received any COVID-19 vaccinations? | - Yes - No   *If Yes, how many doses?*   - One dose - Two doses - Three or more |
| 10 | What type of COVID-19 vaccine(s) you received? | *Open question* |
| **SYMPTOMS**  *Explain to the participant: “I am going to ask you about different symptoms related to COVID-19 illness. It is important that you answer “YES” if these symptoms were new since you were diagnosed with COVID-19 in 2021, or if they occurred within a period of 3 months after infection. If you did not have these symptoms or experienced them before you got COVID-19, answer “NO”.*  *Make a note if the respondent does not remember or does not want to answer the question.* | | |
| 11 | Did you feel fatigue? | - Yes - No |
| 12 | If Yes, how long it lasted? | - Less than 1 month - 1 to 2 months - 2 to 3 months - More than 3 months |
| 13 | Did you feel post-exertional malaise? | - Yes - No |
| 14 | If Yes, how long it lasted? | - Less than 1 month - 1 to 2 months - 2 to 3 months - More than 3 months |
| 15 | Did you had sore throat? | - Yes - No |
| 16 | If Yes, how long it lasted? | - Less than 1 month - 1 to 2 months - 2 to 3 months - More than 3 months |
| 17 | Did you feel muscle pain? | - Yes - No |
| 18 | If Yes, how long it lasted? | - Less than 1 month - 1 to 2 months - 2 to 3 months - More than 3 months |
| 19 | Did you feel dyspnea? (*shortness of breath*) | - Yes - No |
| 20 | If Yes, how long it lasted? | - Less than 1 month - 1 to 2 months - 2 to 3 months - More than 3 months |
| 21 | Did you feel chest pain? | - Yes - No |
| 22 | If Yes, how long it lasted? | - Less than 1 month - 1 to 2 months - 2 to 3 months - More than 3 months |
| 23 | Did you feel joint pain? | - Yes - No |
| 24 | If Yes, how long it lasted? | - Less than 1 month - 1 to 2 months - 2 to 3 months - More than 3 months |
| 25 | Did you feel dry cough? | - Yes - No |
| 26 | If Yes, how long it lasted? | - Less than 1 month - 1 to 2 months - 2 to 3 months - More than 3 months |
| 27 | Did you feel difficulty breathing? | - Yes - No |
| 28 | If Yes, how long it lasted? | - Less than 1 month - 1 to 2 months - 2 to 3 months - More than 3 months |
| 29 | Did you feel diarrhea? | - Yes - No |
| 30 | If Yes, how long it lasted? | - Less than 1 month - 1 to 2 months - 2 to 3 months - More than 3 months |
| 31 | Did you feel anxiety? | - Yes - No |
| 32 | If Yes, how long it lasted? | - Less than 1 month - 1 to 2 months - 2 to 3 months - More than 3 months |
| 33 | Did you feel dizziness? | - Yes - No |
| 34 | If Yes, how long it lasted? | - Less than 1 month - 1 to 2 months - 2 to 3 months - More than 3 months |
| 35 | Did you feel short-term memory loss? | - Yes - No |
| 36 | If Yes, how long it lasted? | - Less than 1 month - 1 to 2 months - 2 to 3 months - More than 3 months |
| 37 | Did you feel mental confusion? | - Yes - No |
| 38 | If Yes, how long it lasted? | - Less than 1 month - 1 to 2 months - 2 to 3 months - More than 3 months |
| 39 | Did you feel loss of attention? | - Yes - No |
| 40 | If Yes, how long it lasted? | - Less than 1 month - 1 to 2 months - 2 to 3 months - More than 3 months |
| 41 | Did you feel difficulty thinking? | - Yes - No |
| 42 | If Yes, how long it lasted? | - Less than 1 month - 1 to 2 months - 2 to 3 months - More than 3 months |
| 43 | Did you feel difficulty in executive function?  *(Difficulty performing daily tasks and work)* | - Yes - No |
| 44 | If Yes, how long it lasted? | - Less than 1 month - 1 to 2 months - 2 to 3 months - More than 3 months |
| 45 | Did you have difficulty in solving problems? | - Yes - No |
| 46 | If Yes, how long it lasted? | - Less than 1 month - 1 to 2 months - 2 to 3 months - More than 3 months |
| 47 | Have you experienced any other symptoms that were not asked about in this questionnaire?  *If Yes, please specify* | Open question |
| *NS- I don't know/can’t tell;*  ***^a^*** *– The English translation was provided by the authors for the purpose of publishing this article. This translation was not submitted to the formal process of translation and cross-cultural adaptation, followed the validation process.* | | |

**Appendix -** Long COVID Questionnaire *(original version in Brazilian Portuguese)*

|  | **Questões** | **Respostas** |
| --- | --- | --- |
| **INFORMAÇÕES GERAIS.**  *Verifique o nome completo e a data de nascimento do participante através de documento de identificação (CPF/RG).* | | |
|  | Data da entrevista | Dia/mes/ano |
|  | Local | Bairro/USF referente |
|  | Nome do entrevistador | Nome codificado |
| 01 | Qual seu nome? | Nome completo |
| 02 | Qual o seu contato? | Colocar DDD e telefone. |
| 03 | Qual o seu endereço? | Endereco completo |
| 04 | Qual sua idade? | Idade/Data de nascimento |
| 05 | Qual seu gênero? | - Masculino |
|  |  | - Feminino |
|  | Se outro, qual? | - Outro |
|  |  | - Preﬁro não dizer |
| 06 | Até que ano você estudou? | - Primário Incompleto |
|  |  | - Primário completo |
|  |  | - Secundário incompleto |
|  |  | - Secundário completo |
|  |  | - Curso Técnico/proﬁssionalizante incompleto |
|  |  | - Curso Técnico/proﬁssionalizante |
|  |  | - Curso superior incompleto |
|  |  | - Curso superior completo |
|  |  | - Pós-graduação incompleta |
|  |  | - Pós-graduação |
|  |  | - NS |
| 07 | Você se considera? | - Branco (a) |
|  |  | - Preto (a) |
|  |  | - Oriental |
|  |  | - Pardo (a) |
|  |  | - Indígena |
|  |  | - NS |
| 08 | Você tinha doenças crônicas pré-existentes ao COVID-19? | - Sim - Não   *Se sim, qual/quais?*  Resposta aberta: |
| 09 | Você se vacinou? | - Sim - Não   *Se sim, com quantas doses?*   - Uma dose - Duas doses - Três ou mais doses |
| 10 | Quais foram as vacinas que você se vacinou? | Resposta aberta: |
| **SINTOMAS.**  *Explique ao participante: “Vou perguntar a você sobre os diferentes sintomas relacionados à doença COVID-19. É importante que você responda "SIM" se esses sintomas eram novos desde que você foi diagnosticado com COVID-19 em ano 2021, ou se os sintomas ocorreram dentro de um período de 3 meses após a infecção. Responda "NÃO" caso você não tinha esses sintomas ou os sentia antes de contrair COVID-19”.*  *Anota caso o entrevistado não se lembre ou não queira responder à pergunta.* | | |
| 11 | Você sentiu fadiga? | - Sim - Não |
| 12 | Se sim, perdurou por quanto tempo? | - Menos de 1 mês - De 1 a 2 meses - De 2 a 3 meses - Mais de 3 meses |
| 13 | Você sentiu mal-estar pós-esforço? | - Sim - Não |
| 14 | Se sim, perdurou por quanto tempo? | - Menos de 1 mês - De 1 a 2 meses - De 2 a 3 meses - Mais de 3 meses |
| 15 | Você sentiu dor de garganta? | - Sim - Não |
| 16 | Se sim, perdurou por quanto tempo? | - Menos de 1 mês - De 1 a 2 meses - De 2 a 3 meses - Mais de 3 meses |
| 17 | Você sentiu dores musculares? | - Sim - Não |
| 18 | Se sim, perdurou por quanto tempo? | - Menos de 1 mês - De 1 a 2 meses - De 2 a 3 meses - Mais de 3 meses |
| 19 | Você sentiu dispneia? (*falta de ar*) | - Sim - Não |
| 20 | Se sim, perdurou por quanto tempo? | - Menos de 1 mês - De 1 a 2 meses - De 2 a 3 meses - Mais de 3 meses |
| 21 | Você sentiu dor no peito? | - Sim - Não |
| 22 | Se sim, perdurou por quanto tempo? | - Menos de 1 mês - De 1 a 2 meses - De 2 a 3 meses - Mais de 3 meses |
| 23 | Você sentiu dores nas articulações? | - Sim - Não |
| 24 | Se sim, perdurou por quanto tempo? | - Menos de 1 mês - De 1 a 2 meses - De 2 a 3 meses - Mais de 3 meses |
| 25 | Você sentiu tosse seca? | - Sim - Não |
| 26 | Se sim, perdurou por quanto tempo? | - Menos de 1 mês - De 1 a 2 meses - De 2 a 3 meses - Mais de 3 meses |
| 27 | Você sentiu diﬁculdade em respirar? | - Sim - Não |
| 28 | Se sim, perdurou por quanto tempo? | - Menos de 1 mês - De 1 a 2 meses - De 2 a 3 meses - Mais de 3 meses |
| 29 | Você sentiu diarreia? | - Sim - Não |
| 30 | Se sim, perdurou por quanto tempo? | - Menos de 1 mês - De 1 a 2 meses - De 2 a 3 meses - Mais de 3 meses |
| 31 | Você sentiu ansiedade? | - Sim - Não |
| 32 | Se sim, perdurou por quanto tempo? | - Menos de 1 mês - De 1 a 2 meses - De 2 a 3 meses - Mais de 3 meses |
| 33 | Você sentiu tontura? | - Sim - Não |
| 34 | Se sim, perdurou por quanto tempo? | - Menos de 1 mês - De 1 a 2 meses - De 2 a 3 meses - Mais de 3 meses |
| 35 | Você sentiu perda de memória de curto prazo? | - Sim - Não |
| 36 | Se sim, perdurou por quanto tempo? | - Menos de 1 mês - De 1 a 2 meses - De 2 a 3 meses - Mais de 3 meses |
| 37 | Você sentiu confusão mental? | - Sim - Não |
| 38 | Se sim, perdurou por quanto tempo? | - Menos de 1 mês - De 1 a 2 meses - De 2 a 3 meses - Mais de 3 meses |
| 39 | Você sentiu perda de atenção? | - Sim - Não |
| 40 | Se sim, perdurou por quanto tempo? | - Menos de 1 mês - De 1 a 2 meses - De 2 a 3 meses - Mais de 3 meses |
| 41 | Você sentiu diﬁculdade de pensar? | - Sim - Não |
| 42 | Se sim, perdurou por quanto tempo? | - Menos de 1 mês - De 1 a 2 meses - De 2 a 3 meses - Mais de 3 meses |
| 43 | Você sentiu diﬁculdade de funcionamento executivo?  *(Diﬁculdade para fazer as tarefas do dia a dia e do trabalho)* | - Sim - Não |
| 44 | Se sim, perdurou por quanto tempo? | - Menos de 1 mês - De 1 a 2 meses - De 2 a 3 meses - Mais de 3 meses |
| 45 | Você sentiu diﬁculdade em resolver problemas? | - Sim - Não |
| 46 | Se sim, perdurou por quanto tempo? | - Menos de 1 mês - De 1 a 2 meses - De 2 a 3 meses - Mais de 3 meses |
| 47 | Você sentiu mais algum sintoma que não foi perguntado neste questionário?  *Se sim, qual/quais?* | Resposta aberta |
| NS-não sabe dizer | | |
